# Retraction Note: Hippocampal neuronal cells that accumulate α-synuclein fragments are more vulnerable to Aβ oligomer toxicity via mGluR5– implications for dementia with lewy bodies

**DOI:** 10.1186/s13024-025-00881-6

**Published:** 2025-08-05

**Authors:** Cassia R Overk, Anna Cartier, Gideon Shaked, Edward Rockenstein, Kiren Ubhi, Brian Spencer, Diana L Price, Christina Patrick, Paula Desplats, Eliezer Masliah

**Affiliations:** 1 Department of Neurosciences, University of California, San Diego, La Jolla, CA USA; 2 Neuropore Therapies, Inc, San Diego, CA 92121 USA; 3 Department of Pathology, University of California, San Diego, La Jolla, CA USA

**Retraction Note**

The Editorial team has retracted this article.

After publication, concerns were raised regarding some of the data presented in the figures. Specifically:

- Panels *Non tg* and *APP tg* in the second row of Fig. 5A appear to overlap;
- Panels *Non tg* in the third rows of Fig. 6B and D appear to overlap;
- Panels *APP tg* and *alpha-syn tg* in the first row of Fig. 7D appear to overlap;
- Panels *Non-tg* in the second rows of Fig. 7B and D appear to overlap;
- Panels *APP tg* and *alpha-syn tg* in the third row of Fig. 7D appear to overlap;
- Some blots in Fig. 9c appear to have similar bands between the two LV-control groups (vehicle and Aβ1–42).

The authors have stated that these errors occurred during figure preparation. However, due to the number of concerns, the Editors-in-Chief no longer have confidence in the presented data.

Cassia R Overk and Eliezer Masliah disagree with this retraction. The other authors have not responded to any correspondence from the editor or publisher about this retraction.

